# Surgical Oncology in Romania: An Analysis of Research and Impact Based on Literature Search in PubMed and Web of Science

**DOI:** 10.1155/2021/5528582

**Published:** 2021-03-08

**Authors:** Sînziana Ionescu, Octavia-Luciana Madge, Ioana Robu, Eugen Brătucu, Claudiu Daha

**Affiliations:** ^1^Oncology Institute Bucharest, First Clinic of General Surgery and Surgical Oncology, 252 Fundeni Road, 2nd District, 022328 Bucharest, Romania; ^2^“Carol Davila” University of Medicine and Pharmacy Bucharest, 37 Dionisie Lupu Street, 1st District, Bucharest, Romania; ^3^University of Bucharest, 5-7 Edgar Quinet Street, 1st District, 010017, Bucharest, Romania; ^4^The Library of the Cluj Medicine and Pharmacy University, 8 Victor Babes Street, 400012 Cluj-Napoca, Romania

## Abstract

**Background:**

With a long tradition and outstanding contributions over time, medical scientific research in Romania has experienced major changes in the last two decades, marked by an increase in scientific publications, originating especially from university centers and fostered by national regulations on publication standards required for professional promotion. This study is aimed at assessing the literature on surgical oncology in Romania, published by Romanian authors in journals indexed in international databases.

**Materials and Methods:**

A literature search was performed, focused on surgical oncology performed in Romania. Two databases, PubMed and Web of Science (WoS), were finally selected and included in the study, which included bibliometric parameters and subject analysis.

**Results:**

The PubMed search retrieved 464,295 articles being published in only 3 Romanian journals, *Chirurgia*, *The Medical-Surgical Journal* (*Iasi*), and *Romanian Journal of Morphology and Embryology*. The search of the Web of Science retrieved 494 records on the subject of surgical oncology in Romania, 449 of which were published after 1989. The 494 articles received 2,102 citations, 4.26 per year, and an overall Hirsch index of 21. Most articles were published in the same 3 Romanian journals as in PubMed. Neoplasms of the digestive system prevailed, followed by articles on general surgical oncology issues, cancer research, and therapy. Bucharest has the highest number of authors, followed by Cluj-Napoca and Iasi.

**Conclusion:**

Research originating from Romania in the field of surgical oncology is present and visible at an international level mainly through Romanian journals. Sustained effort is required from surgical oncology authors to be published in international journals on this subject, as it is the only way to increase global visibility and impact.

## 1. Introduction

According to the World Health Organization, “Cancer is the second leading cause of death globally, being responsible for an estimated 9.6 million deaths in 2018; approximately 70% of deaths from cancer occur in low- and middle-income countries” [1]. Lung, colorectal, gastric, and breast cancers are the most common causes of cancer death worldwide.

Almost a quarter (23.4%) of cancer cases in the world are in Europe, where this disease represents, worldwide, the second leading cause of morbidity and death, with over 4.5 million new cases and over 2 million deaths in 2018 [2].

In Romania, according to the 2019 Romanian Statistical Yearbook, the number of patients classified according to diagnosis at the moment of hospital discharge as cancer was 402,000 in 2018, and the number of new cancer cases declared by family physicians in the same year was 111,000 [3].

Mortality by cancer was the second cause of death in Romania in 2018, with 51,652 deaths, after diseases of the cardiovascular system followed by respiratory system diseases, digestive system diseases, and accidents. Crude death rate by neoplasms per 100,000 inhabitants increased from 181 in 2000 to 232.9 in 2018. According to the official data, the most frequent causes of deaths by neoplasms in 2018 were the neoplasms of the bronchi and lungs (45.3 per 100,000 inhabitants), neoplasm of the colon (18.2 per 100,000 inhabitants), neoplasm of the breast (16 per 100,000 inhabitants), neoplasm of the liver and intrahepatic bile ducts (14.3 per 100,000 inhabitants), and neoplasm of the stomach (14 per 100,000 inhabitants) [4].

The number of cancer cases in the records of the oncology specialists was 490,655 in 2018 and the number of newly diagnosed cancer cases detected in Romania was 61,780, with a territorially uneven distribution and a much higher incidence for the male gender and for the urban environment, and the forecast by 2025 indicates a trend of continuous growth of new cases [5].

The statistics on the total number of surgeries performed in Romania in 2018 for the diagnosis of cancer are not easily available, but there is certainly an increasing number, year by year.

Dissemination of the results of medical research is done firstly by publishing articles in journals and by presenting the results at scientific events. “The results of scientific research are communicated primarily through publications in journals” [6].

However, what is published does not necessarily reflect the interests or needs of patients or professionals in the field, as Lawler et al. noted, referring to the results regarding rarer types of cancer which are not “disseminated as effectively as those for more common cancers” [7].

## 2. Aim

This study is aimed at providing the objective assessment of the literature on surgical oncology in Romania, published by Romanian authors in journals indexed in international databases. The specific goals are to extract information on the evolution of the scientific output in time, the number of authors and their distribution across the large Romanian medical and surgical centers, the impact of published research, the fields of interest within surgical oncology, a basic comparison with neighboring countries, and trends and possible ways to improve global publication and visibility.

## 3. Materials and Methods

The databases (DB) comprised the following: PubMed, Embase, Web of Science (WoS), and Scopus. Google Scholar was also searched, but because of the large record yield, it is very difficult to manage in a structured way and, therefore, was not included in the analysis. Of the four DBs mentioned, two are bibliographic—PubMed and Embase—focusing on subject search, while the other two—Web of Science and Scopus—are bibliometric, focusing on citation and research impact analysis. Given the fact that PubMed and Embase retrieved very similar results (Embase includes MEDLINE, the core component of PubMed), we retained PubMed for the study analysis. In the same way, between WoS and Scopus, the results from WoS (All Databases and Core Collection) were used, mainly because the search results did not show much difference.

Worthy of mention is that Romanian journals not included in these databases were also initially considered for the study, but because of the large proportion (75%) of articles written in Romanian and the almost complete lack of international visibility, the final decision was to exclude them.

### 3.1. PubMed Search

The following search syntax was used: ((((((“Neoplasms/surgery”[**Majr]**) OR (“Neoplasms by site/surgery”[**Majr]**) OR (oncological surgery) OR (surgical oncology))) OR (cancer AND surgical treatment)) OR (tumors AND surgical treatment))) AND ((“Romania”[**Mesh**]) OR (Romania∗[**tiab**])).

It combines index terms (MeSH: Medical Subject Headings) and free text. The initial separate searches of various neoplasms by site, e.g. “colon cancer,” “lung cancer,” and breast cancer,” proved to be impractical because of the difficulties in filtering. Moreover, the search of “Romania” in the field of author affiliation retrieved a large number of articles of surgical oncology in general, but not in Romania, authored and mainly coauthored by Romanians, which were considered irrelevant for the study.

All results were saved to Mendeley, the Elsevier free reference manager, for easy processing and data extraction.

### 3.2. Web of Science Search

The search was performed with the “All Databases” option, which, besides the Core Collection journals (with Impact Factor, indexed in *Journal Citation Reports*), included MEDLINE, BIOSIS, Current Contents, SciELO, Russian SCI, and Derwent Innovation.

The advanced search option was used according to the field syntax, e.g., TS = oncological surgery (search by topic). Boolean combination of terms was performed in the search history option for more accuracy. “Romania” was searched in titles and abstracts and author affiliations.

The retrieved records were submitted to the citation analysis option and saved to an Excel file. Because the “All DB” option did not allow saving of author affiliations, the Core Collection records were extracted from the set for further analysis.

No time limits were defined in the searches, as these could be easily found in the retrieved sets.

## 4. Results

### 4.1. PubMed

**Figure 1:**
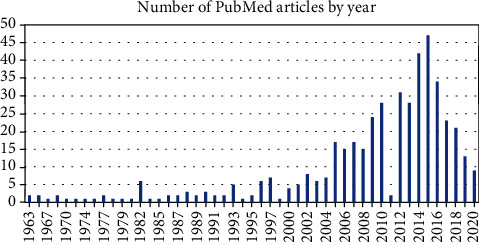
PubMed articles on surgical oncology in Romania by publication year.

At the time of manuscript drafting, the total number of retrieved records was 464. The number of articles per year, as displayed by the PubMed graph, is represented in Figure 1.

The increase in numbers is clearly evidenced starting with the year 2005, reaching a peak in 2015, when 47 articles were indexed.

**Figure 2:**
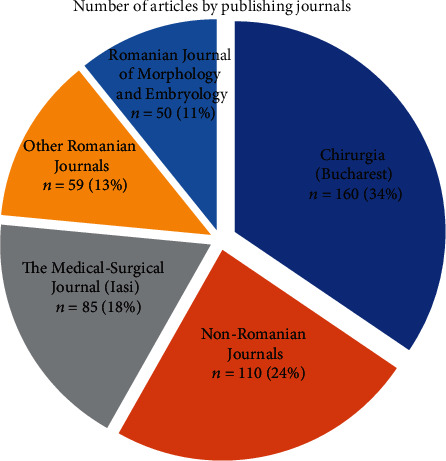
Number of Romanian surgical oncology articles by journal.

The journals in which these articles were published are dominated by the Romanian journals indexed in PubMed, mainly *Chirurgia*, *The Medical-Surgical Journal* (*Iasi*), and *Romanian Journal of Morphology and Embryology*. The number of articles by journal is shown in Figure 2.

Data evidence shows that 295 articles out of 464 were published in only 3 journals, and only less than a quarter of the number of articles was published in non-Romanian journals (110 of 464).

**Figure 3:**
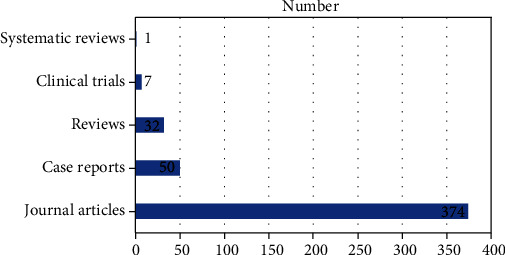
Number of articles by publication type.

According to the PubMed type of articles, the majority are classified as “journal articles,” which we may assume are original research, followed by case reports and reviews, in a significantly smaller number, as shown in Figure 3.

As the PubMed search string allowed a quick comparison of the number of articles in Romania with those in the neighboring countries, a search was performed with the same syntax in which the country name was replaced. Romania is surpassed by Poland with 1,485 articles in surgical oncology and by Hungary with 667. Bulgaria (*n* = 328), Czech Republic (*n* = 271), Croatia (*n* = 152), and Slovakia (*n* = 87) have fewer articles indexed in PubMed on the subject.

### 4.2. Web of Science

**Figure 4:**
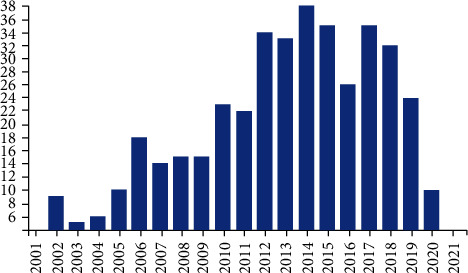
Total number of articles in WoS by year of publication.

**Figure 5:**
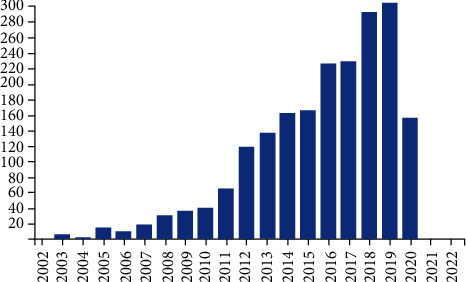
Sum of citations received by year.

The search of WoS All Databases retrieved a total number of 494 records about surgical oncology in Romania, 449 of which were published after 1989. The citation analysis feature of the database evidenced thefollowing information: the total number of 494 received 2,102 citations, which on average means 4.26 per year. Based on these data, the Hirsch index was calculated to be 21. Figures 4 and 5 are extracted as such from the database citation analysis report.

In WoS, a similar search was performed for Poland, retrieving 1,605 records, with an overall Hirsch index of 69, and Hungary, 862 records, with H‐index = 45.

Of the 494 articles, 293 (59.3%) received at least one citation. The highest number of citations received was 54, for an article published in 2009. All cited articles, except 2, were published after 1990.

The journals in which these articles were published maintained the same pattern as in PubMed: 275 articles (55.6%) were published in 3 Romanian journals: *Chirurgia*, *The Medical-Surgical Journal* (*Iasi*), and *Romanian Journal of Morphology and Embryology*. Unlike in PubMed, in the WoS retrieved set, it was readily possible to see the language in which the articles were written. Surprisingly, until as late as 2008, 99% of the articles were written in Romanian. Overall, there were 170 articles (34.4%) written in Romanian of a total of 494.

**Figure 6:**
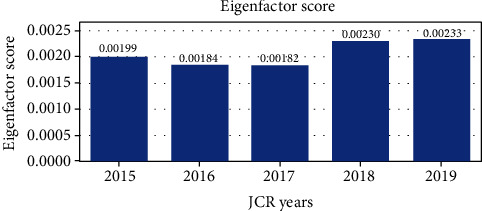
Eigenfactor score of the *Romanian Journal of Morphology and Embryology* for the period 2015-2019.

Other bibliometric parameters would have been of great value in our evaluation, as argued by Roldan-Valadez et al. [8]. However, metrics such as the Eigenfactor score and Article Influence Score proved to be of little relevance for our study, given that they could be retrieved only for 2 journals out of the three significant ones, namely, *Chirurgia* (available for the years 2011-2012!) and *Rom J Morph Embryology* (available for 2010-2019)—the Eigenfactor score of the last available 5 years is shown in Figure 6.

Both the above mentioned journals are in the Q4 (4^th^ quartile) journal rank category, which is also of low significance because they are not in the specific field of surgical oncology.

**Figure 7:**
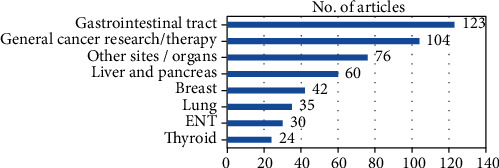
Number of articles by cancer sites and subject focus.

Because the WoS All Databases retrieved set included the largest number of articles, we performed a keyword search on the 494 titles found, the terms used being organs or cancer sites, and correlated them with the “Analyze results” feature of the database. The results are shown in Figure 7.

By far, the majority of articles refer to the digestive system—the gastrointestinal tract, liver, and pancreas, followed by articles on general surgical oncology issues, cancer research, and therapy.

Because one of the objectives of this study was to evaluate the distribution of authors by the most important oncological centers in Romania, the articles indexed in the Core Collection set of the Web of Science were saved separately to an Excel file, because only this database allows saving of author affiliations. The total number of articles found was 259, published between 1977 and 2020, of which 185 were cited at least once.

**Figure 8:**
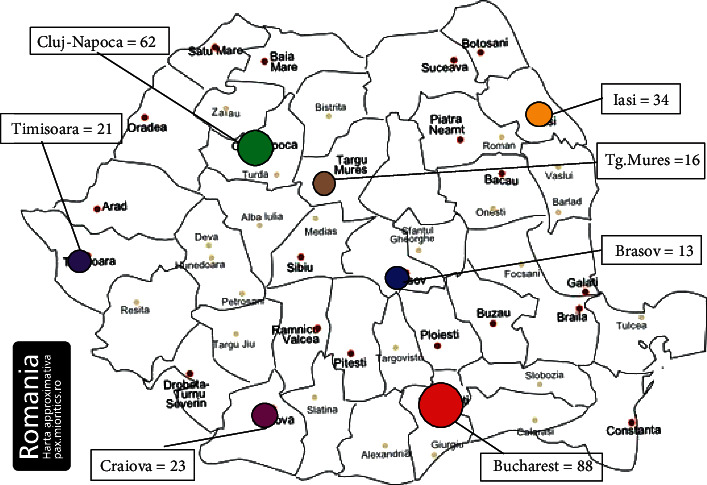
Romania map: geographical distribution of authors.

The geographical distribution of the authors is shown in Figure 8.

As expected, Bucharest has the highest number of authors, followed by Cluj-Napoca and Iasi, the 3 centers being the largest and most renowned in the medical and surgical field.

## 5. Discussion

PubMed is the most comprehensive bibliographic medical database in the world, while Web of Science, as a bibliometric database, is considered the most prestigious when analyzing the quality and impact of research. Our study focused on the analysis of the results from searches on surgical oncology publications from Romania in these two major sources of medical records worldwide.

After 1990, the academic and scientific communities in Romania experienced many changes and new practices, rules, and criteria in all fields of specialization with an opening to new opportunities and research available at the international level.

The last two decades have seen an increase of surgical research originating from Romania, published in journals visible and accessible internationally.

The searches in the two databases returned very close numbers of articles, 464 and 494, and showed an increase in the number of publications starting with 2005-2006.

The first reason for these results is the fact that most articles retrieved are published in three Romanian journals indexed in the databases. The increase in numbers after 2005 can be explained by the fact that the rules and criteria for promotion in higher education in Romania changed along the years. At the same time, journals started to raise the standards and publish research in English to be indexed in international databases, a requirement also taken into consideration in the national rankings.

The fact that only a small number of articles were published in journals from abroad showed that for certain specialties, it is still very difficult for Romanian authors/specialists to get published in the mainstream scientific literature.

The fact that most articles are in the category of journal articles, which we assume to be original research, can be explained by the fact that this type of article is taken into consideration for promotion or as a prerequisite for the PhD thesis.

Compared to the publishing activity in the neighboring East European countries, Romania can be placed on the third place, somewhere in the middle, regarding the number of articles, after Poland, a much larger country, and with more financial resources, and Hungary.

The fact that until very late, only starting with 2008, 99% of the articles were written in Romanian indicated a lower speed of adaptation to international standards. Criteria such as international visibility, which is achieved by publishing in English, were not adopted until imposed at the national level by authorities for promotion in the academic career.

The impact of researching on surgical oncology originating from Romania is illustrated by the number of citations. This increased starting with 2012. A reason for the low number of citations until that moment is the fact that many articles were published in Romanian, which restricted the access of researchers from abroad, doubled by the fact that the three Romanian journals were not very well known abroad or visible at the international level. In addition, the number of articles in the Core Collection was only 259 with only 185 being cited. The Hirsch index value of 21, considerably lower than for Poland or Hungary, is another sign that efforts and improvements are required to raise the visibility and consequently the impact of research in Romania.

One of the problems it seems is the fact that only a few Romanian journals succeeded in being indexed in WoS. Again, in comparison with Poland and Hungary evidence, although these countries have a high number of articles published in their respective national journals, they still have a much better parity with international journals than Romania does.

The analysis of results by subject research areas did not provide any surprise: besides the largest number of digestive cancers prevalent in surgical oncology, the articles focused on general cancer research and therapy, a field yielding the best results for scientific reporting.

**Figure 9:**
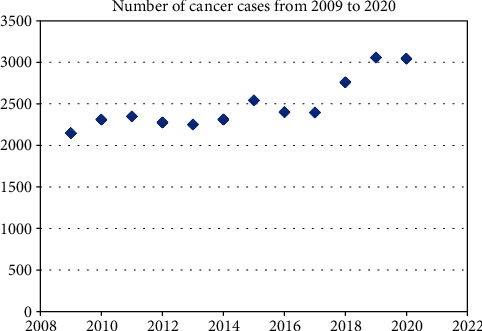
Number of cancer cases diagnosed and treated in general surgery units; the numbers for the year 2020 are those available until the 31th of August.

Furthermore, another area of analysis might be a comparison between the number of PubMed articles and the ascending trend of cancer diagnosis and treatment. For instance, if we are to compare Figures 1 and 9, it can be observed that the year 2015 registered as a peak not only regarding the number of articles on surgical oncology but also due to a noticeable increase in cancer cases [9].

However, in this study, we did not aim to assess the quality of the papers published, unlike in a much similar report from Australia [10].

## 6. Limitations

The limitations of this study are given by the initial preliminary findings, which did not allow the application of more elaborate bibliometrics, because of the scarcity of the results, which did not lend themselves to comparative processing.

## 7. Future Direction

A new education law came into force in 2011 which made it no longer possible to circumvent the imposed criteria. This meant that promotion in the academic career requires more articles published in WoS-indexed journals with an impact factor; this is the most important criterion, overriding publications in other databases. It may further lead to an increase of articles in JCR journals. However, authors should be encouraged to publish in journals of their specialty, which would validate comparative evaluations. As it is now, authors seek publication channels outside their specialty, such as the *Rom J Morph Embryol*, defeating efforts of more precise bibliometric measurements and predictability.

The new criteria adopted by medical universities after the new law came into force also stipulate the position in the list of authors of an article, meaning that one must have, for example, at least 6 articles as first author. The starting point of a future study might be represented by the Romanian authors, similar to the study by LaRocca et al. [11].

## 8. Conclusions

Advanced and high-quality research which matters and brings innovation is published in journals which comply with the highest standards, most often English language journals, indexed in the most important international scientific databases.

Research originating from Romania in the field of surgical oncology is present and visible internationally but still at a low level. For an increase of the impact and visibility of surgical research, potential authors/researchers should consider publishing more in the English language and in international journals which are indexed in prestigious databases. Romanian journals should also increase efforts to raise standards so that journals can be included and indexed in important databases.

In general, Romanian authors of published research are from higher education institutions and have double affiliations—their surgery clinic and a medical school, and they publish as an essential part of their promotional pathway in their academic career.

Future studies could analyze and compare the visibility and impact of research from Romania with those from other countries and analyze the evolution of Romanian research in the general field of surgery.

## Data Availability

We used in our analysis data retrieved from the following databases: PubMed and Web of Science. The results of our searches in these databases were saved to Mendeley, the Elsevier free reference manager, for easy processing and data extraction. The data are available on request from the authors.
